# Aortic Valvuloplasty as Bridging for TAVI in High-Risk Patients with Heyde's Syndrome: A Case Report

**DOI:** 10.1155/2012/946764

**Published:** 2012-08-05

**Authors:** Cosmo Godino, Anna Giulia Pavon, Antonio Mangieri, Alberto Margonato

**Affiliations:** Department of Cardiology, San Raffaele Hospital, Via Olgettina 60, 20131 Milan, Italy

## Abstract

There is a frequent association between aortic valve stenosis and gastrointestinal bleeding, also known as Heyde's syndrome. In these patients, the aortic valve replacement should be recommended as “gold standard.” In high-surgical-risk patients, the Transcatheter Aortic Valve Implantation (TAVI) is an alternative option. However, the risk of bleeding recurrence, related to double antiplatelet therapy started after TAVI, cannot be excluded especially in the first months. We present a case of a patient with a severe aortic valve stenosis and a history of previously documented angiodysplasia and recurrence of gastrointestinal bleeding initially treated only with balloon aortic valvuloplasty that excluded recurrence of bleeding during the subsequent six months of followup. Therefore, a definite transfemoral Edwards XT valve implantation was planned to be performed in case of recurrence of aortic stenosis.

## 1. Introduction

Heyde's syndrome is the association between calcific aortic stenosis (AS) and gastrointestinal (GI) bleeding due to angiodysplasia. It has a major frequency among elderly patients. Alterations in von Willebrand factor due to turbulence across the diseased aortic valve have been incriminated in the pathophysiology of this syndrome. Aortic valve replacement should be recommended as “gold standard.” In these patients, as a surgical intervention results to be a high-risk procedure, Transcatheter Aortic Valve Implantation (TAVI) can be an alternative option. However, the risk of bleeding recurrence related to antiplatelet therapy cannot be excluded.

## 2. Case Summary

**Figure 1:**
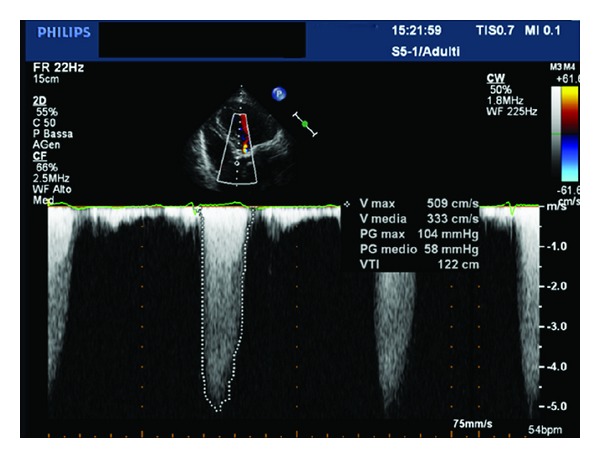
Severe aortic valve stenosis.

**Figure 2:**
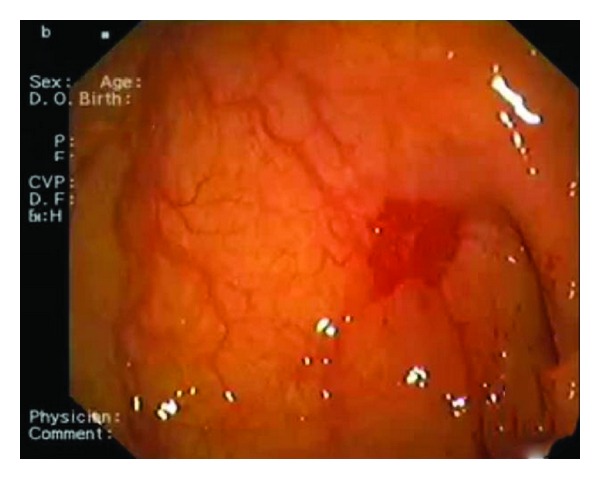
The site of angiodysplasia between the first and the second portion of the duodenum.

**Figure 3:**
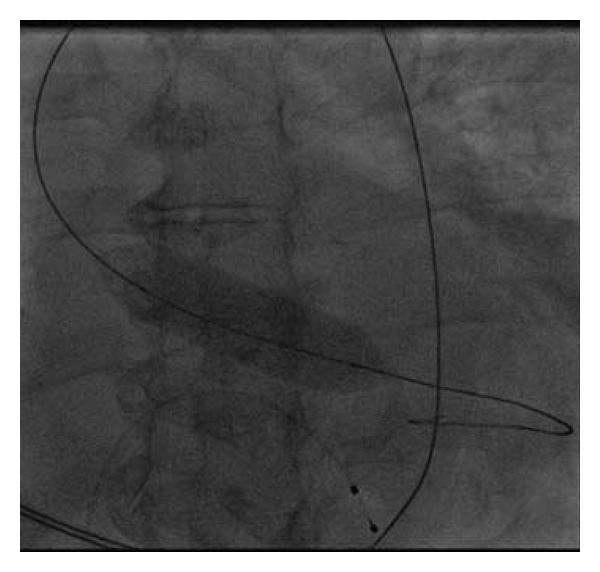
Aortic valvuloplasty.

A 83-year-old woman was referred to our Cardiology Unit for a new onset of severe dyspnea. The clinical history was characterized by mild hypertension, uncomplicated type II diabetes, and hypercholesterolemia. Even more importantly, recurrence of anemization, requiring for two times blood transfusion, was noticed in the last eight months. Her physical examination showed a blood pressure of 90/60 mmHg, pulse rate of 97 beats/min, and an O_2_ saturation of 97%. A midsystolic rough 3/6 murmur was present at the second right intercostal space irradiating toward the neck. No abnormalities were found at the pulmonary examination. Signs of left ventricular hypertrophy with an ST depression in V3–V6 were present at the ECG. Considering the significant ejection rough murmur a transthoracic echocardiogram was performed that revealed the presence of severe degenerative aortic valve stenosis (mean gradient 58 mmHg, maximum gradient 104 mmHg, aortic valve area 0.7 cm^2^) (Figure 1). A moderate depression of the left ventricular systolic function (LVEF 41%) with a symmetrical hypertrophy (interventricular septum 12 mm, posterior wall 13 mm) was also reported. Moreover, the laboratory findings showed severe iron deficiency anemia (hemoglobin 6.1 g/dL, hematocrit 19%). Severe iron deficiency anemia was confirmed collecting new blood test and subsequent transfusion of 2 units of packed red blood cells was done with significant improvements in haematologic status. An esophagogastroduodenoscopy (EGDS) was performed to clarify the cause of anemization. A 5 mm slightly elevated angiodysplasia was found between the first and the second portion of the duodenum (Figure 2). An argon plasma coagulation laser was used to treat the lesion, then prophylactic high dose of oral pantoprazole was given to reduce risk of new bleeding. On the basis of the instrumental findings the diagnosis of Heyde's syndrome was suggested. In order to evaluate the feasibility of an aortic valve substitution, standard and logistic EuroSCORE were calculated and found to be, respectively, 12 and 35%. Considering the high-risk clinical profile, in accordance with the cardiac surgeon, the patient was screened for TAVI. However, taking into account the risk of bleeding recurrence related to the dual antiplatelet therapy that should be started after TAVI we preferred, in the first instance, to perform only a balloon aortic valvuloplasty (BAV). The aortic valvuloplasty was successfully performed 15 days after hospitalization (Figure 3) with a significant mean aortic gradient reduction from 58 mmHg to 22 mmHg, without any GI bleeding until the discharge. After three months of clinical followup, a significant improvement in dyspnea was noted and no recurrence of GI bleeding was reported together with stable values of haemoglobin in two subsequent controls. The echocardiogram performed at six-month followup showed the persistent of the acute BAV result with mean aortic gradient of 27 mmHg, jet velocity 3.4 m/sec, and aortic valve area 1.2 cm^2^. Therefore, we decided to postpone definitive Edward XT (23 mm) valve implantation in case of recurrence of severe aortic stenosis.

## 3. Discussion

There's a frequent association between aortic valve stenosis and gastrointestinal bleeding, also known as Heyde's syndrome. Severe aortic valve stenosis remains the main cause of morbidity and mortality in the elderly, reaching a prevalence of 2–7% above the age of 65 years old. According to different sources, between 7 and 29% of patients with diagnosed GI bleeding angiodysplasia suffer from aortic valve stenosis or aortic sclerosis and 3% of advanced aortic valve stenosis patients have gastrointestinal bleeding [1]. A possible explanation calls into question a high shear stress induced by the stenotic valve orifice that leads to a conformational change in the structure of large multimers of von Willebrand factor making it more prone to proteolysis by ADAMTS 13. The reduced number of high-molecular-weight (HMW) multimers impairs platelet-mediated hemostasis and predisposes patient to gastrointestinal bleeding [2]. There are many methods of therapy; however aortic valve replacement (AVR) should be recommended as “gold standard” [3]. This should act in reducing the shear stress caused by a narrowed aortic valve orifice, preventing the reduction of HMW multimers and so GI bleeding. In patients with high surgical risk, the Transcatheter Aortic Valve Implantation (TAVI) can be an alternative option, however, the risk of bleeding recurrence related to single and also more to double antiplatelet therapy (usually prescribed after TAVI) cannot be excluded especially in the first months after procedure. In this case a patient with a severe aortic valve stenosis and history of angiodysplasia with recurrences of GI bleeding was treated at the beginning only with BAV that excluded recurrence of GI bleeding during the subsequent three months of followup. We believe that this type of step-TAVI approach is useful in patients with the Heyde's syndrome in which the double antiplatelet therapy may predispose GI bleeding in the subsequent months. In this case the CoreValve was not considered because of the need of six months of dual antiplatelet therapy against the three months required after an Edward valve implantation. Finally, the advantage of this step-TAVI approach must be balanced with the risk of periprocedural BAV-related adverse events reported in up to 15% of cases.

## 4. Conclusion

Heyde's syndrome should be suspected in patients with GI bleeding and calcific severe AS. In these patients the risk of bleeding recurrence, related to double antiplatelet therapy started after TAVI, cannot be excluded especially in the first months. Therefore, a step-TAVI procedure with aortic balloon valvuloplasty performed firstly must be conserved as a good strategy in case of high-surgical-risk patients with Heyde's syndrome.
